# Genetic dynamics underlying phenotypic development of biomass yield in triticale

**DOI:** 10.1186/1471-2164-15-458

**Published:** 2014-06-10

**Authors:** Wenxin Liu, Manje Gowda, Jochen C Reif, Volker Hahn, Arno Ruckelshausen, Elmar A Weissmann, Hans Peter Maurer, Tobias Würschum

**Affiliations:** Crop Genetics and Breeding Department, China Agricultural University, 100193 Beijing, China; State Plant Breeding Institute, University of Hohenheim, 70599 Stuttgart, Germany; Leibniz Institute of Plant Genetics and Crop Plant Research (IPK), 06466 Gatersleben, Germany; Competence Centre of Applied Agricultural Engineering COALA, University of Applied Sciences Osnabrück, 49076 Osnabrück, Germany; Saatzucht Dr. Hege GbR Domäne Hohebuch, 74638 Waldenburg, Germany

**Keywords:** Dynamic QTL, Precision phenotyping, Biomass, Triticale, BreedVision

## Abstract

**Background:**

The nature of dynamic traits with their phenotypic plasticity suggests that they are under the control of a dynamic genetic regulation. We employed a precision phenotyping platform to non-invasively assess biomass yield in a large mapping population of triticale at three developmental stages.

**Results:**

Using multiple-line cross QTL mapping we identified QTL for each of these developmental stages which explained a considerable proportion of the genotypic variance. Some QTL were identified at each developmental stage and thus contribute to biomass yield throughout the studied developmental phases. Interestingly, we also observed QTL that were only identified for one or two of the developmental stages illustrating a temporal contribution of these QTL to the trait. In addition, epistatic QTL were detected and the epistatic interaction landscape was shown to dynamically change with developmental progression.

**Conclusions:**

In summary, our results reveal the temporal dynamics of the genetic architecture underlying biomass accumulation in triticale and emphasize the need for a temporal assessment of dynamic traits.

**Electronic supplementary material:**

The online version of this article (doi:10.1186/1471-2164-15-458) contains supplementary material, which is available to authorized users.

## Background

Quantitative trait locus (QTL) mapping approaches are popular genomic tools to dissect the genetic architecture underlying complex traits and to identify QTL [1]. The basis for these approaches are mapping populations, which are commonly phenotyped at only a single time point. It is well known, however, that many traits of agronomic importance are under the control of complex dynamic regulation [2]. Consequently, the traditional static examination completely neglects the developmental dynamics underlying trait formation. For example, biomass changes with time and thus, two genotypes can be phenotypically identical at a certain developmental stage while the temporal patterns of genetic control may vary between them. Until now, only little is known about the genetic dynamics of complex traits. Yan et al. [3] evaluated plant height in rice at different developmental stages and observed some QTL that could be detected at all stages whereas others were only detectable at one or some of them. Busemeyer et al. [4] recently reported a dynamic mapping for biomass in triticale and also observed developmental stage specific QTL.

In order to assess the temporal changes in the genetic control of trait formation, the phenome of the plants must be assessed at several time points. A key component for monitoring the phenotypic changes of plants is the development of appropriate phenotyping technologies which for crops must enable phenotyping under field conditions [5, 6]. Busemeyer et al. [7] have recently described the development of the ‘BreedVision’ precision phenotyping platform for non-invasive, high-throughput and high-dimensional phenotyping of small grain cereals under field conditions. This platform incorporates light curtains, laser distance sensors, a 3D-Time-of-Flight camera and hyperspectral imaging and traits are predicted based on sensor fusion, i.e., the combination of sensors and their information. This platform is of particular interest as it enables the evaluation of traits not amenable to traditional phenotyping and in addition, can replace destructive measurements thereby enabling multiple measurements of the same plants. Biomass, for example, is traditionally evaluated by harvesting yield plots with a field chopper. The major disadvantage of this approach is, that it is destructive, thus prohibiting the assessment of other traits as well as the biomass development of the plots over time. The precision phenotyping platform has been calibrated for biomass yield of triticale which yielded a high prediction accuracy and heritability for the predicted biomass [4].

Triticale (× *Triticosecale* Wittmack L.) shows a broad variation for biomass yield [8] and is therefore well suited to study the genetics underlying this trait in small grain cereals. A powerful approach for QTL mapping is to employ multiple segregating families [9, 10]. Busemeyer et al. [4] used biomass data from four families predicted based on measurements of the precision phenotyping platform at three developmental stages in combination with an association mapping approach to detect QTL. Association mapping is an identity-by-state approach and the employed biometric model has been shown to be rather conservative [11]. Consistenly, the analysis of part of these data with an identity-by-descent approach identified substantially more QTL [12]. The aim of this study was therefore to reanalyse the triticale biomass data [4] employing an identity-by-descent approach to assess the temporal dynamics of QTL contributions to biomass yield. Here, we describe the detection of main effect QTL for biomass yield in triticale at three different developmental stages, the variation in the contribution of these QTL to the genotypic variance over time, and the dynamics of epistatic QTL contributing to the genetic architecture underlying phenotypic development of biomass yield.

## Results

**Figure 1:**
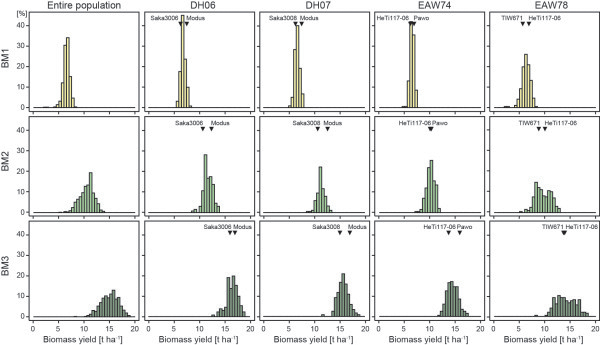
**Phenotypic development of biomass yield.** Histograms of the phenotypic values of biomass yield at three developmental stages (BM1-BM3) for the entire population and for each of the four families (DH06, DH07, EAW74, EAW78).

In the entire mapping population with 647 DH lines derived from four families, we observed significant (*P* < 0.01) genotypic variances  and genotype-by-environment interaction variances  for biomass yield at all three developmental stages (BM1 – BM3) (Additional file 1: Table S1). The ratio of  to  ranged from approximately 3:1 for BM1 to 7:1 for BM2. The heritabilities were high and ranged from 0.81 for BM1 to 0.91 for BM2. Phenotypic correlations between biomass yield at the three developmental stages were 0.84 for BM1-BM2, 0.89 for BM2-BM3 and 0.75 for BM1-BM3 (all significant at *P* < 0.01). The phenotypic values of the parents differed to varying degrees ranging from Δ0.4 to 1.2 for BM1, from Δ0.0 to 1.7 for BM2 and from Δ0.1 to 1.9 for BM3 (Figure 1). Orthogonal contrasts of the means of the families and their respective parents were not significant except for family EAW78 for BM3 (*P* < 0.05). The trait distributions approximately followed a normal distribution except for family EAW78 for which the distribution at BM2 and BM3 became bimodal. The variation within each family increased with increasing developmental stage (Additional file 1: Table S1, Figure 1) and in each family DH lines transgressed the respective parents for all three developmental stages. Taken together, the data set captures the progression of biomass yield over time and is therefore well suited to study the underlying genetic dynamics.

**Figure 2:**
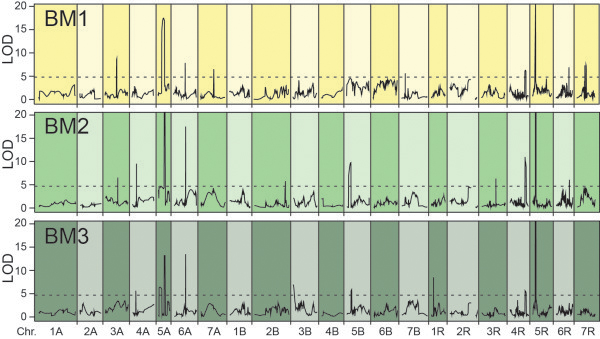
**Main effect QTL.** QTL for biomass yield detected at three developmental stages (BM1-BM3).

**Figure 3:**
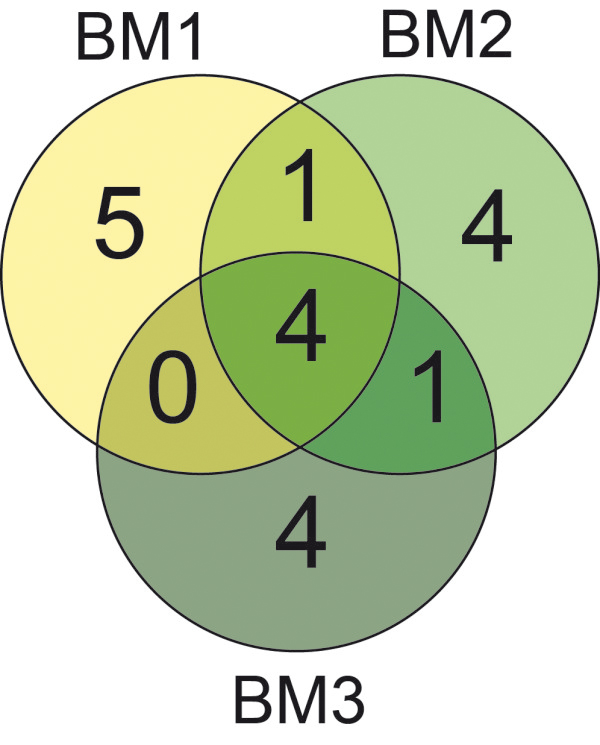
**Specific and overlapping QTL.** Venn diagram for biomass yield QTL detected at three developmental stages (BM1-BM3).

**Figure 4:**
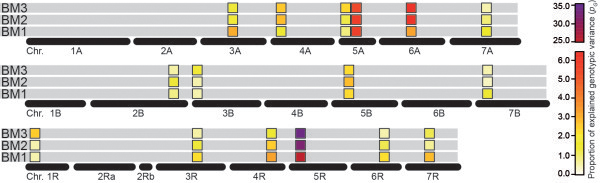
**Temporal dynamics of QTL.** Temporal development of the contribution of the QTL detected for any of the three developmental stages (BM1-BM3) to the proportion of explained genotypic variance for biomass yield.

**Figure 5:**
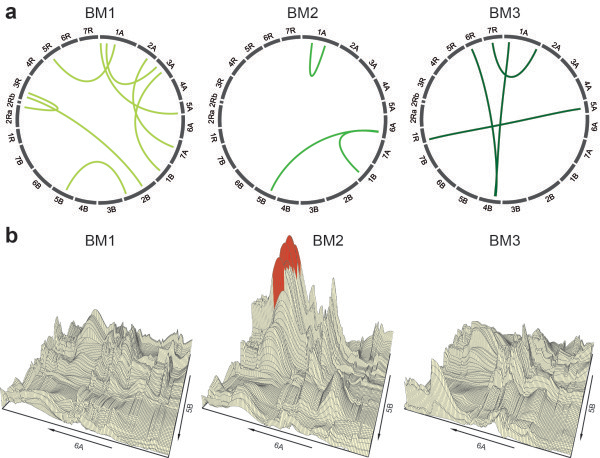
**Epistatic QTL. (a)** Epistatic QTL for biomass yield at three developmental stages (BM1-BM3). **(b)** Temporal development of the epistatic interaction landscape for the QTL detected at BM2 involving chromosomes 6A and 5B.

Multiple-line cross QTL mapping revealed 10 QTL for BM1 and BM2 and 9 QTL for BM3 (Table 1, Additional file 1: Table S2, Figure 2). Interestingly, we observed biomass QTL that were detected at all three developmental stages as well as QTL that were only detected at one or two of the developmental stages (Figure 3). Combined, the QTL explained between 62.2 (BM1) and 64.7 (BM2) percent of the genotypic variance. The proportion of genotypic variance explained by single QTL ranged from 2.5 to 28.0 for BM1, from 1.7 to 34.0 for BM2 and from 2.2 to 35.6 percent for BM3 (Additional file 1: Table S2). The major QTL detected at all three developmental stages was located on chromosome 5R. Another QTL which consistently contributed more than 5 percent to the genotypic variance was identified on chromosome 5A and on chromosome 6A a QTL contributing a similar proportion to BM2 and BM3 was found. We used fivefold cross-validation to assess the quality of the obtained QTL results (Table 1). This revealed a bias in the estimation of the proportion of genotypic variance which cross-validated still ranged between 34.0 (BM1) and 44.8 (BM2) percent. The QTL frequency distributions revealed some QTL that were detected in a high number of runs whereas other QTL identified with the full data set were only detected in few runs (Additional file 1: Figure S1). By contrast, few QTL were detected in a considerable number of runs which have not been identified in the full data set (e.g., BM2 QTL on chromosome 7A).We next assessed the contribution to the genotypic variance at BM1, BM2 and BM3 for all loci detected as QTL for any of the three developmental stages. This substantiated the presence of chromosomal regions which stably contribute to biomass yield as opposed to regions which only contribute to biomass yield in a temporally restricted manner (Figure 4).The full 2-dimensional epistasis scan revealed epistatic QTL for all three developmental stages (Figure 5a). Eight epistatic QTL were identified for BM1, three for BM2 and four for BM3. The contribution of these epistatic QTL to the genotypic variance was small and ranged between 1.0 and 3.5 percent. The epistatic interaction landscape undergoes temporal changes with development as illustrated in Figure 5b for the QTL detected at BM2 involving chromosomes 6A and 5B.

**Table 1:** **Results of QTL mapping and fivefold cross-validation**

	BM1	BM2	BM3^§^
QTL_DS_	10	10	9
*p*_G-DS_	62.2	64.7	62.8
QTL_ES_	7.1	9.4	7.3
*p*_G-ES_	55.8	64.0	57.0
*p*_G-TS_	34.0	44.8	38.2
Relative bias	39.1	30.0	33.0

Number of detected QTL (QTL_DS_), proportion of genotypic variance (in percent) explained by the detected QTL across all families in the data set (*p*_G-DS_), proportion of genotypic variance averaged over estimation sets (*p*_G-ES_) and averaged over test sets (*p*_G-TS_), and relative bias (%) in the estimation of *p*_G_. ^§^ reported in Alheit et al. [12].

## Discussion

Many traits of biological or agronomic importance undergo dynamic changes with time and the developmental progression of the individuals. It therefore appears obvious that these changes will at least in part be reflected by similar changes in the underlying genetics. However, a temporal assessment of the genetic architecture of complex traits has thus far largely been neglected. In this study we therefore performed a dynamic mapping of biomass yield QTL in triticale to decipher the genetic dynamics underlying phenotypic development of this trait.

### Phenotypic development of biomass yield

A prerequiste for efficient QTL detection in multiple families is the precise estimation of phenotypic values [13]. In addition, for traits which are traditionally assessed by destructive measurements, alternative non-invasive approaches are required to enable monitoring dynamic changes of the trait over time. In this study we focused on biomass yield as the accumulation of biomass is central to agricultural productivity, employing triticale as model crop for small grain cereals. The earliest developmental stage (BM1) was chosen at BBCH stage 49 as this represents the earliest time point when triticale would be harvested for biomass. Biomass was predicted based on sensor measurements of the precision phenotyping platform and we observed high heritabilities for all three developmental stages at which biomass yield was assessed. This in combination with the high prediction accuracies underlines the great potential of precision phenotyping, especially for a temporal assessment of dynamic traits.

Biomass yield was characterized by a progression with time and the development of the plants (Figure 1). The correlations of biomass yield at the different time points were highest for the directly successive time points (BM1-BM2, BM2-BM3) and decreased with increasing temporal and thus developmental distance between time points (BM1-BM3). The variation in phenotypic values increased with development of the plants (Figure 1, Additional file 1: Table S1). This does not appear to be caused by an increased influence of the environment as the contribution of the genotype-by-environment interaction variance was strongest at BM1. Rather, the effects of the genetic factors contributing to biomass yield become more pronounced the longer they act on the trait. This is exemplified by the development of biomass in family EAW78. Gowda et al. [8] have recently shown that grain yield, heading time, spikes per square meter, 1000-kernel weight, and early plant height are key contributors to early biomass yield. Family EAW78 segregates for a major plant height QTL which likely corresponds to the rye dwarfing gene *Ddw1* and which causes the observed bimodal distribution [12]. However, the distribution of the biomass yield values in family EAW78 shows a strong temporal plasticity. At BM1 the biomass values are normally distributed, then turn into the bimodel distribution at BM2 which at BM3 becomes less pronounced. Thus, *Ddw1* appears to have the strongest contribution to biomass yield at BM2 after which its influence on biomass declines or that of other factors becomes more prominent. This illustrates the plasticity of the trait and the temporal contribution of genetic factors to its expression and highlights the need for a temporal assessment of the underlying genetics.

### Detection of biomass yield QTL

Busemeyer et al. [4] used an association mapping approach to analyze the data but identified only the major QTL on chromosomes 5A and 5R. By contrast, the linkage mapping approach applied here appears more powerful with regard to QTL detection in this data set as we identified nine or ten QTL for biomass yield at all three developmental stages. These QTL were supported by the QTL frequency distributions (Table 1, Figure 2, Additional file 1: Figure S1). Consequently, the proportion of genotypic variance (*p*_*G*_) explained by the QTL detected here was approximately twice as high as that described in the previous study. For all three developmental stages the *p*_*G*_ was around 60 percent and even cross-validated still amounted to a considerable 30 to 39 percent (Table 1). This is in accordance with the quantitative nature of the trait which implies many QTL with effects too small to be detected in QTL mapping given a reasonable population size.

### Contribution of epistasis to biomass yield

Epistasis refers to interactions between the alleles at two or more genetic loci [14]. It contributes to the genetic architecture of many complex traits and has recently been reported for different crops including maize, wheat and rapeseed [10, 13, 15–17]. The orthogonal contrasts between family means and the means of the respective parents were only significant for BM3 in family EAW78 which indicates the presence of epistasis. It must be noted however, that non-significant orthogonal contrasts do not imply abscence of epistasis. Our analysis revealed epistatic QTL for all three developmental stages (Figure 5). The detection power for epistatic QTL, however, more strongly depends on the population size than that for the detection of main effect QTL. Thus, despite the relatively large mapping population employed here, many epistatic QTL may have remained undetected due to insufficient QTL detection power. In contrast to the main effect QTL for which similar numbers were detected for each developmental stage, we identified substantially more epistatic QTL for BM1 as compared to BM2 and BM3. This corroborates previous findings from Busemeyer et al. [4] and suggests a varying contribution of epistasis to the genetic architecture of biomass yield over time. The proportion of genotypic variance explained by these epistatic QTL was rather small and averaged 2.4, 1.2 and 1.5 percent for BM1, BM2 and BM3, respectively. Nevertheless, this result illustrates the contribution of epistasis to the genetic architecture of biomass yield, especially assuming that a number of epistatic QTL had remained undetected.

### Temporal genetic patterns of biomass regulation

The nature of dynamic traits with their temporal changes suggests at least some plasticity in the underlying genetic control. We observed an almost equal number of QTL for biomass yield for each of the three developmental stages but the Venn diagram indicated both specific and overlapping QTL (Figures 2 and 3). The detection of a QTL at all three time points indicates that it stably contributes to biomass yield throughout the studied development, ranging from the stage where the awns are just visible to very early dough development. By contrast, some QTL were only detected at one or two of these developmental stages. Two QTL were identified at two developmental stages which in both cases were successive stages, i.e., BM1 and BM2 or BM2 and BM3. For each developmental stage we identified four or five QTL that were only detected for that particular stage. As illustrated by the LOD profiles (Figure 2) this does not appear to be caused by peaks close to the significance threshold, being by chance slightly above or below it for the different time points. By contrast, it appears to reflect the dynamic genetics underlying biomass accumulation in triticale. This is supported by the variable contribution of the loci detected for any of the three developmental stages to the genotypic variance at BM1, BM2 and BM3, respectively (Figure 4). Interestingly, we observed a similar plasticity for the epistatic interactions suggesting that the entire genetic architecture of biomass yield undergoes dynamic changes during the developmental progression of the plants.

## Conclusions

In this study, we employed phenomics data of biomass yield generated at three developmental stages by precision phenotyping of a large mapping population of triticale. We show the phenotypic plasticity of this trait and demonstrate that this is reflected in a similar plasticity of the underlying genetics. Thus, the genetics of dynamic traits should best be assessed in a temporal manner to capture all the genetic factors that contribute to the trait during development.

## Methods

### Plant material, field trials and phenotypic data

The plant material, field trials and the collection of phenotypic data used in this study have been described by Busemeyer et al. [4]. In brief, phenotypic data for biomass yield were obtained by non-invasive prediction based on a precision phenotyping platform [7]. A calibration experiment was performed based on 25 diverse triticale genotypes at three developmental stages: BM1 = BBCH stage 49 (awns visible), BM2 = BBCH 69 (late flowering), and BM3 = BBCH 81 (very early dough development) [18]. The plants were harvested with a field chopper to determine reference fresh weight based on which the calibration models were established. With these calibration models biomass yield was predicted at the three developmental stages in a mapping population consisting of 647 doubled haploid (DH) [19, 20] triticale lines. The results for BM3 have in part been reported by Alheit et al. [21]. The population consisted of four families designated DH06 (131), DH07 (120), EAW74 (200), and EAW78 (196) which have been described by Alheit et al. [12]. The DH lines were grown in partially replicated designs [22] including common checks with 960 plots per location, at two locations in two years. Phenotypic data were analyzed by ordinary lattice analysis of variance [23]. Variance components were determined by the restricted maximum likelihood (REML) method assuming a full random model and heritability (*h*^*2*^) on an entry-mean basis was estimated from the variance components as the ratio of genotypic to phenotypic variance [24]. Best linear unbiased estimates (BLUEs) were estimated across environments assuming fixed effects for the genotype. All statistical analyses were performed using ASReml 3.0 [25].

### Multiple-line cross QTL mapping

The DH lines were genotyped with DArT markers and QTL mapping was done based on the integrated consensus linkage map described by Alheit et al. [12]. For QTL mapping, an additive genetic model was chosen and the joint analysis across families was performed with a model assuming specific QTL effects for every family (disconnected model) [26] as described in detail by Steinhoff et al. [9]. In brief, the multiple-line cross QTL mapping model was:

where Y was a *N* × 1 column vector of the BLUE values of phenotypic data of *N* progenies coming from *P* families. J was a *N* × *P* matrix whose elements were 1 or 0 according to whether or not individual *i* belonged to family *p* and *M* was a *P* × 1 vector of family specific means. X_q_ (X_c_) a *N* × *P* matrix containing the expected number (ranging from 0 to 2) of allele *k* for each individual in family *p* at QTL *q* (cofactor *c*), and B_q_ (B_c_) was a *P* × 1 vector of the expected allele substitution effects of QTL *q* (cofactor *c*) in family *p*. ϵ was the vector of the residuals.

Cofactor selection was performed using PROC GLMSELECT implemented in the statistical software SAS [27]. The presence of a putative QTL in an interval was tested using a likelihood-ratio test with the statistical software R [28]. LOD-thresholds of 4.8 for BM1, 4.6 for BM2 and 4.6 for BM3 were used corresponding to an experiment-wise type I error of *P* < 0.10, based on 2000 permutations [29]. Cofactors were excluded within a distance to the marker interval under consideration smaller than 10 cM and the support interval of a QTL was defined as a LOD fall-off of 1.0 expressed as position on the chromosome in centimorgans (cM) [30]. The proportion of genotypic variance explained by the detected QTL was estimated as *R*^*2*^_*adj*_/*h*^*2*^[31]*.* Biomass yield QTL were declared as overlapping between the three developmental stages if they fell within an arbitrarily defined 10 cM interval surrounding the QTL. Fivefold cross-validation was done as described by Liu et al. [32].

The epistasis scan for pairwise interactions was done with the model described above which was extended by the term X_*q’*_B_*q’*_ for the second locus and the interaction term between the two loci *q* and *q’* X_*qq’*_B_*qq’*_. We used an α-level of 0.05 and followed the suggestion of Holland et al. [33] dividing the α-level by the number of possible independent pairwise interactions between chromosome regions, assuming two separate regions per chromosome (*P* < 5.3e-5). The circular plots illustrating the epistatic interactions were created with Circos [34]. The detected epistatic QTL were illustrated by these circular plots (Figure 5a) showing the interactions among different chromosome regions. The temporal development of one QTL detected at BM2 involving chromosomes 6A and 5B was illustrated by the 3-dimensional interaction landscape between these two chromosomes. In Figure 5b the –log_10_(*P* values) of all tested pairwise interactions were plotted and consequently the higher the peak, the stronger the association of the epistatic interaction with the trait. Significant interactions are marked in red.

## Electronic supplementary material

Additional file 1: Table S1: Summary statistics for biomass yield [t ha-1] at the three developmental stages. **Table S2.** QTL detected for biomass yield at three developmental stages (BM1-BM3). **Figure S1.** QTL frequency distributions. (PDF 50 KB)

## References

[1] Würschum T (2012). Mapping QTL for agronomic traits in breeding populations. Theor Appl Genet.

[2] Wu R, Lin M (2006). Functional mapping – how to map and study the genetic architecture of dynamic camplex traits. Nat Rev Genet.

[3] Yan J, Zhu J, He C, Benmoussa M, Wu P (1998). Molecular dissection of developmental behaviour of plant height in rice (Oryza sativa L.). Genetics.

[4] Busemeyer L, Ruckelshausen A, Möller K, Melchinger AE, Alheit KV, Maurer HP, Hahn V, Weissmann EA, Reif JC, Würschum T (2013). Precision phenotyping of biomass accumulation in triticale reveals temporal genetic patterns of regulation. Sci Rep.

[5] Montes JM, Melchinger AE, Reif JR (2007). Novel throughput phenotyping platforms in plant genetic studies. Trends Plant Sci.

[6] White JW, Andrade-Sanchez P, Gore MA, Bronson KF, Coffelt TA, Conley MM, Feldmann KA, French AN, Heun JT, Hunsaker DJ, Jenks MA, Kimball BA, Roth RL, Strand RJ, Thorp KR, Wall GW, Wang G (2012). Field-based phenomics for plant genetics research. Field Crops Res.

[7] Busemeyer L, Mentrup D, Möller K, Wunder E, Alheit K, Hahn V, Maurer HP, Reif JC, Würschum T, Müller J, Rahe F, Ruckelshausen A (2013). Breedvision - A multi-sensor platform for non-destructive field-based phenotyping in plant breeding. Sensors (Switzerland).

[8] Gowda M, Hahn V, Reif JC, Longin CFH, Alheit KV, Maurer HP (2011). Potential for simultaneous improvement of grain and biomass yield in Central European winter triticale germplasm. Field Crops Res.

[9] Steinhoff J, Liu W, Maurer HP, Würschum T, Longin FH, Ranc N, Reif JC (2011). Multiple-line cross quantitative trait locus mapping in european elite maize. Crop Sci.

[10] Steinhoff J, Liu W, Reif JC, Della Porta G, Ranc N, Würschum T (2012). Detection of QTL for flowering time in multiple families of elite maize. Theor Appl Genet.

[11] Würschum T, Liu W, Gowda M, Maurer HP, Fischer S, Schechert A, Reif JC (2012). Comparison of biometrical models for joint linkage association mapping. Heredity.

[12] Alheit KV, Busemeyer L, Liu W, Maurer HP, Gowda M, Hahn V, Weissmann S, Ruckelshausen A, Reif JC, Würschum T (2013). Multiple-line cross QTL mapping for biomass yield and plant height in triticale (x *Triticosecale* Wittmack). Theor Appl Genet.

[13] Liu W, Reif JC, Ranc N, Porta GD, Würschum T (2012). Comparison of biometrical approaches for QTL detection in multiple segregating families. Theor Appl Genet.

[14] Carlborg Ö, Haley CS (2004). Epistasis: Too often neglected in complex trait studies?. Nat Rev Genet.

[15] Buckler ES, Holland JB, Bradbury PJ, Acharya CB, Brown PJ, Browne C, Ersoz E, Flint-Garcia S, Garcia A, Glaubitz JC, Goodman MM, Harjes C, Guill K, Kroon DE, Larsson S, Lepak NK, Li H, Mitchell SE, Pressoir G, Peiffer JA, Rosas MO, Rocheford TR, Romay MC, Romero S, Salvo S, Villeda HS, Da Silva HS, Sun Q, Tian F, Upadyayula N (2009). The genetic architecture of maize flowering time. Science.

[16] Reif JC, Maurer HP, Korzun V, Ebmeyer E, Miedaner T, Würschum T (2011). Mapping QTLs with main and epistatic effects underlying grain yield and heading time in soft winter wheat. Theor Appl Genet.

[17] Würschum T, Maurer HP, Dreyer F, Reif JC (2013). Effect of inter- and intragenic epistasis on the heritability of oil content in rapeseed (Brassica napus L.). Theor Appl Genet.

[18] Lancashire PD, Bleiholder H, van Boom TD, Langelüddeke P, Stauss R, Weber E, Witzenberger A (1991). A uniform decimal code for growth stages of crops and weeds. Annals of Applied Biology.

[19] Würschum T, Tucker MR, Reif JC, Maurer HP (2012). Improved efficiency of doubled haploid generation in hexaploid triticale by in vitro chromosome doubling. BMC Plant Biol.

[20] Würschum T, Tucker MR, Maurer HP (2014). Stress treatments influence efficiency of microspore embryogenesis and green plant regeneration in hexaploid triticale (×Triticosecale Wittmack L.). In Vitro Cell Dev Biol Plant.

[21] Alheit KV, Reif JC, Maurer HP, Hahn V, Weissmann EA, Miedaner T, Würschum T (2011). Detection of segregation distortion loci in triticale (x Triticosecale Wittmack) based on a high-density DArT marker consensus genetic linkage map. BMC Genomics.

[22] Williams E, Piepho H-P, Whitaker D (2011). Augmented p-rep designs. Biom J.

[23] Cochran WG, Cox GM (1957). Experimental Designs.

[24] Melchinger AE, Utz HF, Schön CC (1998). Quantitative trait locus (QTL) mapping using different testers and independent population samples in maize reveals low power of QTL detection and larger bias in estimates of QTL effects. Genetics.

[25] Gilmour AR, Gogel BG, Cullis BR, Thompson R (2009). ASReml user Guide Release 3.0. VSN International Ltd, Hemel Hempstead.

[26] Blanc G, Charcosset A, Mangin B, Gallais A, Moreau L (2006). Connected populations for detecting quantitative trait loci and testing for epistasis: An application in maize. Theor Appl Genet.

[27] (2008). SAS/STAT 9.2 User's guide.

[28] (2010). R: a language and environment for statistical computing.

[29] Doerge RW, Churchill GA (1996). Permutation tests for multiple loci affecting a quantitative character. Genetics.

[30] Lander ES, Botstein S (1989). Mapping mendelian factors underlying quantitative traits using RFLP linkage maps. Genetics.

[31] Utz HF, Melchinger AE, Schön CC (2000). Bias and sampling error of the estimated proportion of genotypic variance explained by quantitative trait loci determined from experimental data in maize using cross validation and validation with independent samples. Genetics.

[32] Liu W, Maurer HP, Reif JC, Melchinger AE, Utz HF, Tucker MR, Ranc N, Della Porta G, Würschum T (2013). Optimum design of family structure and allocation of resources in association mapping with lines from multiple crosses. Heredity.

[33] Holland JB, Portyanko VA, Hoffmann DL, Lee M (2002). Genomic regions controlling vernalization and photoperiod responses in oat. Theor Appl Genet.

[34] Krzywinski M, Schein J, Birol I, Connors J, Gascoyne R, Horsman D, Jones SJ, Marra MA (2009). Circos: An information aesthetic for comparative genomics. Genome Res.

