# Supplementary material for: Genetic dynamics underlying phenotypic development of biomass yield in triticale
Source: BMC Genomics. 2014 Jun 10;15(1):458. doi: 10.1186/1471-2164-15-458 (PMC4070554; doi:10.1186/1471-2164-15-458)
Supplement: Supplementary file 1 — Additional file 1: Table S1: Summary statistics for biomass yield [t ha-1] at the three developmental stages. Table S2. QTL detected for biomass yield at three developmental stages (BM1-BM3). Figure S1. QTL frequency distributions. (PDF 50 KB) [file 12864_2013_6128_MOESM1_ESM.pdf]

Genetic dynamics underlying phenotypic development of biomass yield in triticale

Wenxin Liu[1], Manje Gowda[2], Jochen C. Reif[3], Volker Hahn[2], Arno Ruckelshausen[4], Elmar A. Weissmann[5], Hans Peter Maurer[2], Tobias Würschum[2§]

Additional files

Additional file 1 – Additional Tables and Figures.

Table S1 - Summary statistics for biomass yield [t ha^{-1}] at the three developmental stages.

	BM1	BM2	BM3§
Min	2.27	5.16	8.13
Mean	6.52	10.51	14.97
Max	8.10	13.77	18.71
σ_G^2	2.91**	16.83**	23.28**
σ_{GxE}^2	0.95**	2.32**	4.39**
σ_e^2	2.69	6.00	17.34
h^2	0.81	0.91	0.85

Genotypic variance (σ_G^2), genotype by location interaction variance (σ_{GxE}^2), error variance (σ_e^2), and heritability (h^2). ** significant at the 0.01 probability level. § reported in Alheit et al. 2013

Table S2 - QTL detected for biomass yield at three developmental stages (BM1-BM3).

	Chromosome	Position in cM (support interval)	p_G
BM1			
	3A	57.6 [55.9 - 57.7]	4.0
	5A	26.7 [21.7 - 32.3]	8.2
	6A	57.7 [57.5 - 57.9]	3.4
	7A	66.0 [65.0 - 66.8]	2.8
	7B	19.6 [19.6 - 19.6]	2.5
	4R	79.6 [75.9 - 81.7]	2.8
	5R	14.4 [13.8 - 16.3]	28.0
	6R	65.2 [65.0 - 65.3]	3.1
	7R	40.7 [40.7 - 40.7]	3.2
	7R	46.6 [46.2 - 48.4]	3.3
BM2			
	3A	60.4 [59.9 - 60.5]	2.1
	4A	20.5 [20.4 - 20.7]	2.9
	5A	32.8 [29.3 - 33.6]	7.3
	6A	57.7 [57.5 - 57.9]	5.9
	2B	151.1 [151.1 - 151.8]	1.8
	5B	20.4 [13.9 - 20.8]	2.9
	3R	70.4 [70.4 - 70.8]	1.7
	4R	74.9 [74.9 - 77.0]	3.5
	5R	15.7 [15.0 - 17.0]	34.0
	6R	65.2 [65.0 - 65.3]	1.9
BM3			
	4A	17.1 [16.8 - 17.8]	2.2
	5A	8.6 [3.3 - 17.3]	2.6
	5A	32.8 [28.3 - 33.6]	5.5
	6A	57.7 [57.5 - 57.9]	5.5
	3B	0.0 [0.0 - 3.8]	2.5
	5B	24.3 [20.8 - 24.8]	2.6
	1R	10.6 [10.2 - 10.6]	3.3
	4R	75.4 [74.9 - 81.2]	2.2
	5R	14.4 [13.8 - 16.3]	35.6

Chromosome, position with support interval and proportion of genotypic variance explained by the QTL (in percent).

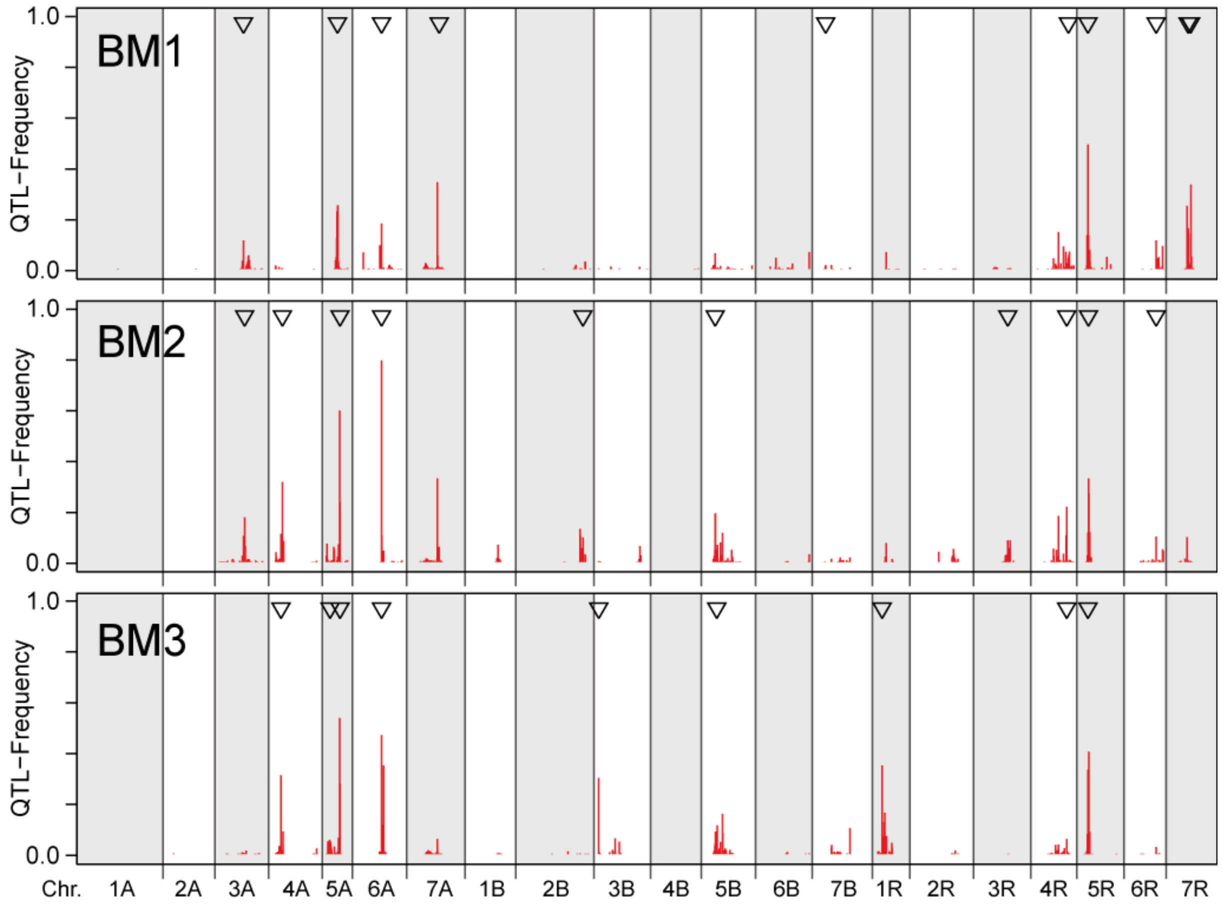

Figure S1 - QTL frequency distributions.

Frequency distributions for the QTL detected at three developmental stages (BM1-BM3) derived from fivefold cross-validation. The arrowheads indicate QTL positions of the full data set.
